# The impact of cycling on the physical and mental health, and quality of life of people with disabilities: a scoping review

**DOI:** 10.3389/fspor.2024.1487117

**Published:** 2025-01-06

**Authors:** Nina Mosser, Glen Norcliffe, Annika Kruse

**Affiliations:** ^1^ Department of Human Movement Science, Sport and Health, University of Graz, Graz, Austria; ^2^ Faculty of Environmental and Urban Change, York University, Toronto, ON, Canada

**Keywords:** impairment, disability, adaptive cycles, bicycling, health, mobility

## Abstract

Adaptive cycling holds potential for promoting physical and mental health among individuals with disabilities, who often face barriers to traditional cycling and other forms of exercise. This scoping review systematically examines existing scientific literature to assess the effects of adaptive cycling on the physical and mental health of individuals with disabilities. Following a widely recognized methodological scoping review framework, 35 qualitative and quantitative studies were identified through comprehensive database searches and manual screenings. The review highlights the positive impacts of adaptive cycling on cardiovascular fitness, muscle strength, and overall physical well-being, as well as improvements in mental health and quality of life. Despite these benefits, significant research gaps remain, particularly concerning adaptive cycling modalities, such as sociable cycles, chair transporters, and power-assisted bikes, which were underrepresented in the existing literature. This review underscores the need for further studies to provide a comprehensive understanding on the effects of different adaptive cycling modalities. Such studies are essential to improve accessibility and ultimately support the health and social inclusion of individuals with disabilities.

## 1 Introduction

Cycling is widely acknowledged to have numerous health benefits including the enhancement of cardiovascular fitness, muscle strength, joint mobility, proprioception and mental health (1, 2). Regular participation in cycling can significantly reduce the risk of chronic diseases including cardiovascular diseases, metabolic diseases, and certain cancers (1, 2). Moreover, cycling supports weight management, usually reduces stress levels, and contributes to overall well-being. Its low-impact nature makes it an ideal form of exercise for individuals across various age groups and fitness levels, further solidifying its role as a valuable health-promoting activity (1, 2).

Despite the well-documented benefits of cycling, not everyone can engage in this form of activity. Individuals with certain disabilities may face significant barriers that limit their ability to participate in traditional cycling (3). As stated by the World Health Organization, about 1.3 billion people, or approximately 16% of the world's population, live with some form of disability (4). According to the United Nations, persons with disabilities “include those who have long-term physical, mental, intellectual, or sensory impairments which in interaction with various barriers may hinder their full and effective participation in society on an equal basis with others” [(5), p. 4]. This definition includes physical, sensory, cognitive, and developmental disabilities, affecting individuals across all age groups (4). The prevalence of disabilities is expected to increase as the population ages, with elderly individuals experiencing higher rates of physical impairment (6).

For people with disabilities, an inability to engage in regular physical activity, such as cycling, can lead to a range of health risks (7). High levels of sedentary behavior are common among this population, which can result in secondary health conditions including obesity, cardiovascular disease, and mental health issues such as depression and anxiety (8, 9). These risks underscore the importance of promoting accessible forms of physical activity to maintain health and prevent disease in individuals with disabilities.

Adaptive cycling plays a crucial role in addressing these challenges. By incorporating various technical adaptations—such as hand cycles for those with lower limb impairments, tricycles for enhanced stability and hemiplegia, tandem bicycles for individuals with visual impairments, specially adapted cycles for persons with skeletal dysplasia, and electrical-assisted features to accommodate varying levels of physical capability—adaptive cycling makes it possible for individuals with a wide range of disabilities to participate (3, 10). These innovations enable people with disabilities to enjoy the physical and psychological benefits of cycling, while promoting inclusion and active living (3, 10). Given these considerations, adaptive cycles present a distinctive opportunity to promote physical activity among individuals with disabilities, helping to mitigate sedentary behavior and the associated health risks.

While various forms of adaptive physical activity have been reviewed in the literature (11, 12), adaptive cycling has not yet received the same level of attention. This form of exercise may offer distinct benefits due to its low-impact nature, its potential for fostering social inclusion, and assumed adaptability to a wide range of impairments. A scoping review focused on adaptive cycling is thus necessary to address the specific needs, outcomes and possibilities associated with this activity. This review will not only summarize the current body of research but will also highlight critical gaps in knowledge and suggest directions for future research in this underexplored area.

The purpose of this scoping review is to systematically investigate the existing scientific literature. The guiding research question is: “Is there scientific information available about the effects of adaptive cycling on the physical and mental health of individuals with disabilities?” By synthesizing the available evidence, this review seeks to (1) highlight the potential benefits and limitations of adaptive cycling, (2) identify research gaps, and (3) suggest directions for future studies. The findings will provide valuable insights for healthcare providers, policymakers, and individuals with disabilities.

## 2 Methods

We adhered to the methodological framework for scoping reviews outlined by Arksey and O'Malley (13), which consists of five key stages: (1) identifying the research question; (2) identifying relevant studies; (3) study selection; (4) charting the data; and (5) collating, summarizing, and reporting the results. We note that steps 4 and 5 were combined for efficacy, without compromising the quality of the analysis.

In addition, we adhered to the Preferred Reporting Items for Systematic Reviews and Meta-Analyses extension for Scoping Reviews (PRISMA-ScR) guidelines (14) throughout the review process, ensuring transparency and methodological rigor.

### 2.1 Identifying the research question

As outlined above, this scoping review is guided by the following research question: “Is there scientific information available about the effects of adaptive cycling on physical and mental health of individuals with disabilities?” Thus, the aim of the following steps is to synthesize existing research to understand the influence of adaptive cycling on the health of individuals with disabilities and to identify any research gaps.

### 2.2 Identifying relevant studies

To identify relevant studies, we conducted a literature search in April 2024 using three databases: PubMed, Scopus, and Web of Science. We performed a spot-check of two other databases (Cochrane and ScienceDirect) which resulted in no further identification of new, significant literature. No publication date restrictions were applied to ensure a comprehensive search, capturing all studies written in English or German available up to the search date. The search terms were developed based on the PICO framework (15), focusing on Population, Intervention, and Outcome, while the Comparison component was excluded to maximize the inclusion of all potential studies. The search was conducted in a two-step process:

1. Initially, we conducted searches in each of the three databases using a primary search term based on the research question of our study, focusing on the Title/Abstract fields, as shown in Table 1.
2. Subsequently, we conducted a further search using a secondary search term, also outlined in Table 1. This secondary term was developed based on the articles identified in the initial search. Specifically, we revisited the search using terms related to adaptive cycles that had already been identified. This step was taken to ensure that we did not overlook any specific designations or variations of adaptive cycles. The aim was to further refine the search results.

**Table 1:** Final primary and secondary search term used for the identification of relevant studies.

Primary search term (title/abstract)
(cycling OR bike*OR biking OR bicycl* OR “adaptive cycling”) AND (disabilit* OR amputee* OR wheelchair* OR para-* OR impairment*) AND (physical OR mental OR physiological OR psychological OR health OR “quality of life”) NOT (animal* OR menstr* OR “life cycle”)
Secondary search term (title/abstract)
(framerun* OR racerun* OR “frame running” OR “race running” OR “frame runner” OR “race runner” OR handbik* OR handcycl* OR (hand AND (bicycle OR bike OR cycl*)) OR (tandem AND (bicycle OR bike OR cycl*)) OR tricycle OR quadricycl* OR (stationary AND (bicycle OR bike OR cycle)) OR e-bike OR “power-assisted bike”) AND (disabilit* OR amputee* OR wheelchair* OR para-* OR impairment*) AND (physical OR mental OR physiological OR psychological OR health OR “quality of life”) NOT (animal* OR menstr* OR “life cycle”)

**Figure 1:**
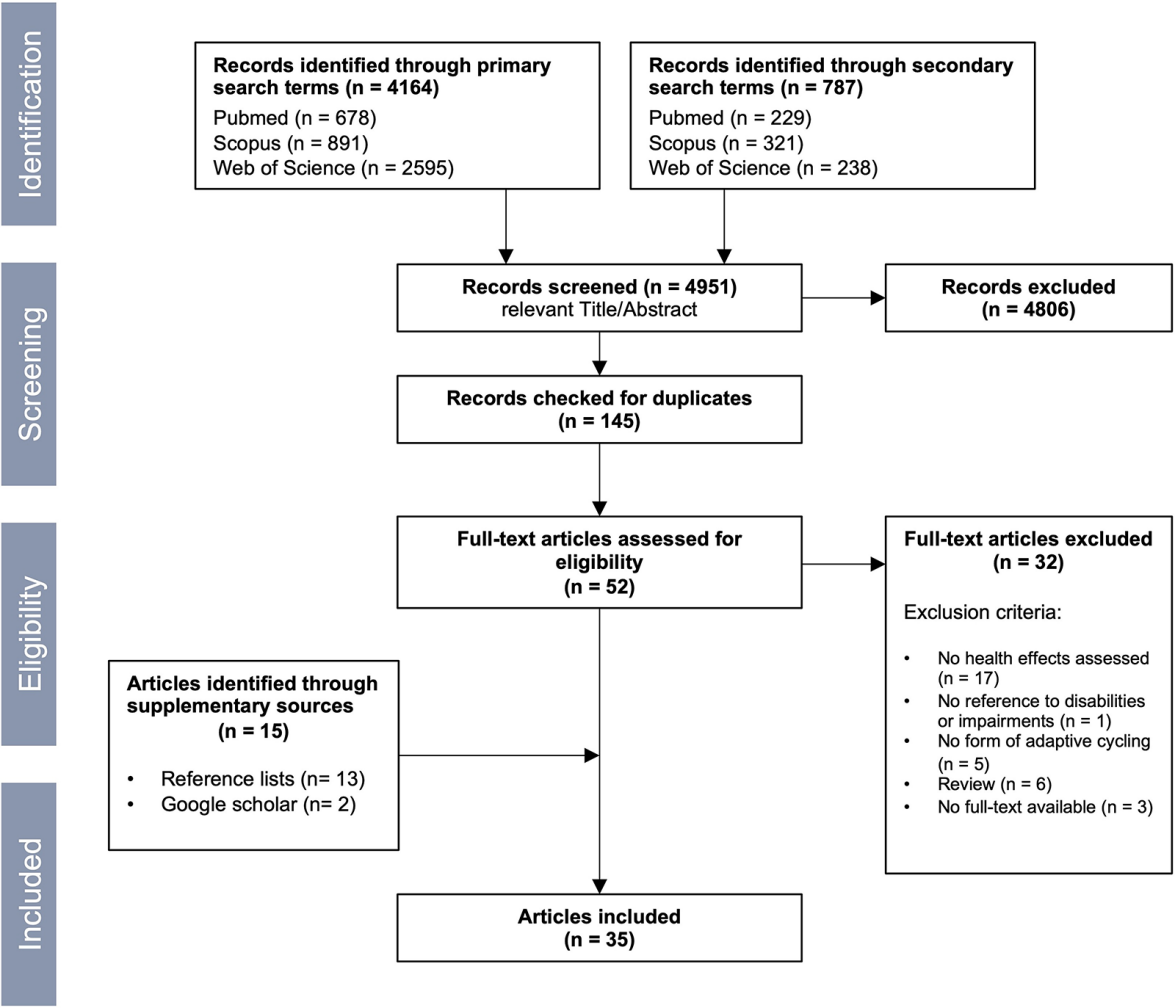
PRISMA flow chart for article selection.

In addition to this two-step process, we conducted supplementary searches by reviewing the reference lists of identified articles and utilizing Google Scholar to identify any additional relevant sources. The final search terms, which were adjusted according to the requirements of each respective database, are presented in Table 1. For more information on the results, please refer to Figure 1.

### 2.3 Study selection

The study selection process was based on the PRISMA flow chart model (16). Following primary and secondary searches, a manual preselection of relevant studies was conducted based on their title and abstract. Subsequently, duplicates were removed, and another screening of the remaining articles was performed using the inclusion and exclusion criteria outlined in Table 2. During the review of these articles, reference lists were also examined for additional relevant literature. Additionally, Google Scholar was screened for further relevant articles. The study selection was initially conducted by one primary reviewer based on the inclusion and exclusion criteria defined beforehand by all authors. For the final selection, all authors were involved in reviewing the articles to ensure consensus. Any conflicts or disagreements that arose during the process were resolved through discussion among the authors. Only articles that met the inclusion criteria after thorough examination were included into the analysis. After agreement among the authors on the final selection of studies, one reviewer processed the findings. The final selection of articles then underwent the steps outlined in Chapter 2.4.

**Table 2:** Overview of inclusion and exclusion criteria applied during the manual preselection and subsequent screening of articles identified in the primary and secondary literature searches.

Inclusion criteria	Exclusion criteria
• Health effects assessed • Any kind of disability or impairment • All forms of adaptive cycling • Empirical studies • Full-text available (except conference papers) • Full-text in English or German • Grey literature if relevant	• No health effects assessed • No reference to disability or impairment • No form of adaptive cycling • Review (direct inclusion of relevant studies) • No full-text available (except conference papers)

### 2.4 Charting data and collating, summarizing and reporting results

The previously selected studies were systematically organized in a table (see Table 3), wherein various characteristics of each study were collated. These characteristics included the reference (main author and publication year), study design, study participants, number of participants, type of adaptive cycle used, intervention employed, intervention groups, and identified health outcomes. Subsequently, an overview was generated based on this table, reflecting the current body of literature along with its gaps. Afterwards, a synthesis of the compiled data was conducted to provide a cohesive summary of the findings. This synthesis aimed to elucidate the significance and implications of the identified research outcomes, shedding light on major trends, patterns, and areas that require further investigation. In addition, particular attention was paid to placing the results into the broader context of adaptive cycling research in order to gain valuable insights for practitioners and researchers in this field.

**Table 3:** Key characteristics of the selected studies.

Reference	Study design	Participants	Number of participants	Groups	Adapted bicycle	Intervention	Health outcomes
Abel et al. (17)	Observational study	Spinal cord injury, amputation of both legs	27	Wheelchair racing (*n* = 10) handbiking (*n* = 17)	Handbikes and wheelchair racer	Basal metabolism evaluation, incremental exercise test, endurance test	Energy expenditure high enough to maintain fitness, may prevent cardiovascular diseases
Bakkum et al. (18)	Experimental study	Inactive people with long-term spinal cord injury	20	Hybrid cycling (*n* = 10) handbiking (*n* = 10)	Hybrid cycle, handbike	30 min per day, 2×/week for 16 weeks	Improvement in cardiovascular fitness
Blumenstein et al. (19)	Experimental study	Healthy subject	1	–	Adapted e-bike	Single bouts of exercise	Improvement in space orientation and allows tuning of the electric motor's power to meet individual physical needs
Bryant et al. (20)	Experimental study	Children with cerebral palsy	15	Spastic bilateral CP (*n* = 11) dyskinetic CP (*n* = 4)	Frame runner	3×/week for 12 weeks	Enjoyment, increased standing ability, no change in the CP QoL-Child questionnaire scores, significant improvement of bone density
Daly et al. (21)	Experimental study	Children with cerebral palsy	3	–	Adaptive bicycles	30 min daily	Significant improvement on the energy expenditure index, improvement in gross motor function (all subjects)
Fowler et al. (22)	Experimental study	Children with cerebral palsy	62	Cycling (*n* = 31) control (*n* = 31)	Stationary bicycle	30 sessions over 12 weeks	Significant improvements in locomotor endurance, gross motor function, and some measures of strength
Fu et al. (23)	Observational study	Elderly with physical disability	41	–	Cycling wheelchair	30 min per day, 5×/week for 4 weeks	Improvement in quality of life and aerobic capacity
Gervasoni et al. (24)	Experimental study	People wih multiple sclerosis	20	Crossover: group A (*n* = 10) group B (*n* = 10)	Arm cycling and tailored task-oriented exercise	20 sessions over 8 weeks	Reduction in fatigue and motor fatigability, increase in finger movement rate
Grecco et al. (25)	Experimental study	Unilateral transtibial amputees	34	Non-athlete untrained (*n* = 17) Paralympic athletes (*n* = 17)	Stationary bicycle and resistance training	3×/week for 8 weeks	Improvement in general functional condition, muscle strength, and cardiorespiratory performance
Hjalmarsson et al. (26)	Experimental study	Adolescents and young adults with cerebral palsy	15	–	Frame runner	2×/week for 12 weeks	Increase in cardiorespiratory endurance, increase in thickness of medial gastrocnemius muscle and decreased ankle dorsiflexion on more-affected side, increase in passive hip flexion on less-affected side
Hoekstra et al. (27)	Observational study	Wheelchair users	59	–	Handbike	4 months free-living condition	POpeak, VO2peak and waist circumference improved significantly
Hussein et al. (28)	Experimental study	Children with hemiplegic cerebral palsy	48	Study (*n* = 24) control (*n* = 24)	Arm cycling	30 min arm cycling and 60 min gait training exercises over a 6 month period	Significant improvement in arm swing, significant increase in flexion angular displacements of the hip and ankle joints during gait cycle
Inckle (29)	Descriptive study	People with physical disability, impairment or mobility impairment	7	–	Standard two-wheeled bicyle, handbike, trike, recumbent	Experience of cycling from ten to more than 50 years	Experience of mobility, independence, and freedom, huge benefits for mental and physical health and wellbeing
Kamelska et al. (30)	Observational study	Visually impaired and properly sighted people	26	Visually impaired (*n* = 13) properly sighted (*n* = 13)	Tandem bicycle	1.5–2.5 h per day, 3–5×/week for 7 months	Statistically significant increases in VO2max and Pmax, no time × visual impairment interaction effect was found
Kamelska et al. (31)	Observational study	Visually impaired and properly sighted people	26	Visually impaired (*n* = 13) properly sighted (*n* = 13)	Tandem bicycle	1.5–2.5 h per day, 3–5x/week for 7 months	Similar improvement in majority of hemodynamic variables, visual impairment did not limit health benefits of regular physical activity
Kim et al. (32)	Experimental study	Chronic stroke patients	32	Experimental (*n* = 16) control (*n* = 16)	Stationary bicycle	30 min per day, 5×/week for 6 weeks	Significant improvements in balance and gait abilities, improvements in balance, 10-m walking test score (gait) significantly greater in cycling group
Kim et al. (33)	Experimental study	People with a spinal cord injury	15	Exercise (*n* = 8) control (*n* = 7)	Indoor handbike	60 min per day, 3×/week for 6 weeks	Compared to control group significantly decreased BMI, fasting insulin, and HOMA-IR levels, and significantly increase in Vo2peak and strength in shoulder abduction, adduction, flexion, and extension and elbow flexion and extension
King et al. (34)	Experimental study	Children with cerebral palsy	7	–	Hip-extensor tricycle	10 weeks daily	Visually analysed gait improved, but hip estensor strength did not, childrens and parental reports on use and enjoyment were positive
Kouwijzer et al. (35)	Observational study	People with health conditions such as spinal cord injury, amputation, or multiple trauma history	136	–	Handbike	5 months training	Life satisfaction increased, mental health showed no change over time, improvement in cardiorespiratory fitness was associated with an increase in life satisfaction
Kouwijzer et al. (36)	Observational study	People with health conditions like spinal cord injury	143	–	Handbike	5 months training and 1 year follow-up	Body satisfaction significantly increased during training period and decreased at follow-up, improvements in physical capacity and waist circumference significantly associated with improvements in body satisfaction
Lauhoff et al. (37)	Experimental study	People with Parkinson's disease	23	–	Stationary bicycle	30 min per day, 1x/week for 6 weeks	Statistically significant improvements noted in balance, activities of daily living and mobility, trend towards improvement for exercise tolerance, no significant effect on QoL
Leblanc et al. (38)	Experimental study	Children with cerebral palsy	7	–	Bicyle, tricycle	10 sessions over 5 weeks	No significant difference in gross motor function and locomotor endurance, significant improvement of locomotor performances of lying and reversal motor capacities, significant improvement in the locomotor performance in daily life reported by parents
Lousada et al. (39)	Observational study	People with cerebral palsy	5	–	Frame runner	3 different sprint training sessions	Frame running at a sufficient intensity to promote health and fitness adaptations possible
Mayo et al. (40)	Experimental study	people within 12 months of acute Stroke who were able to walk >10 m independently	87	Cycling (*n* = 43) exercise (*n* = 44)	Stationary bicycle	15–30 min cycling per day vs. disability-targeted exercises for 12 months	Both programs were equally effective in maintaining walking capacity after discharge from stroke rehabilitation
McGough et al. (41)	Experimental study	People with mild to moderate Parkinson's disease	41	–	Tandem bicycle	3×/week for 10 weeks	Statistically significant physical performance improvement across domains of gait, balance, and mobility
Pickering et al. (42)	Observational study	Children with cerebral palsy	25	–	Adaptive bicycles	6 weeks of adaptive cycling	Enjoyment of this experience, improved sense of well-being
Shafizadeh et al. (43)	Observational study	Racerunning athletes	8	–	Frame runner	Series of 100 m sprints on frame runner	Racerunning athletes with neurological motor disorders absorb the impact shock of framerunning through strategy that mimics able-bodied runners
Stone et al. (44)	Observational study	Competitive and recreational handcyclists	13	Competitive (*n* = 7) recreational (*n* = 6)	Handbike	Bouts of exercise at training (50% POpeak), competition (70% POpeak), and sprint intensity	Greater flexibility in the thorax, shoulders, and scapula in the competitive group, indicating that kinematic adaptations attributable to technical training potentially optimize muscle recruitment and force generation of the arm
Valent et al. (45)	Observational study	People with a recent spinal cord injury	162	Handcycling (*n* = 55) non-handcycling (*n* = 82) not recorded (*n* = 25)	Handbike	Regular rehabilitation program	Significantly larger increase in Vo(2)peak, POpeak, and elbow extension strength in subjects with paraplegia, no influence on any outcome measures in postrehabilitation period
Valent et al. (46)	Experimental study	People with tetraplegia	22	–	Handbike	24 sessions within 8–12 weeks	Significant improvements in POpeak, Vo(2)peak, mechanical efficiency, and shoulder abduction strength
Valent et al. (47)	Experimental study	People with a spinal cord injury	40	Experimental (*n* = 20) control (*n* = 20)	Handbike	30–45 min per day, 2×/week for 9–39 weeks	Strong tendencies for improvement in wheelchair capacity(POpeak and oxygen pulse), significant effects on shoulder exo- and endo-rotation and unilateral elbow flexion strength, no improvements on pulmonary function
van der Linden et al. (48)	Observational study	Frame running athletes	115	–	Frame runner	3 months of frame running	Subjects felt increased muscle stretch and self-confidence, some had extreme fatigue or sore muscles after training, less out of breath during mobility tasks and felt improved functional mobility, some reported increased muscle tightness and some a Frame Running-related injury lasting more than 4 weeks
van Schie et al. (49)	Observational study	Young athletes with mobility limitations	62	–	Frame runner	Minimum of 3 months of frame running	Significant positive change on all three subscales of the PIADS questionnaire, most change experienced in performance, the ability to participate, happiness and self-confidence, increased QoL
Vogt et al. (50)	Experimental study	Adolescents with intellectual and developmental disabilities	11	Crossover	Stationary bicycle	10 min moderate cycling	Temporarily enhances neuronal activity in relation to cognitive performance
Williams and Pountney (51)	Experimental study	Non-ambulant children with CP	11	ABA design with participants acting as their own controls	Adapted static bicycle	3×/week for 6 weeks	Improvements in functional ability

#### 2.4.1 Definition of health categories

To ensure clarity in the presentation and interpretation of results, it is important to define the categories of health outcomes discussed in this review. In the following, physical health refers to improvements in physiological functions such as cardiovascular fitness, strength, and endurance (52), while mental health is conceptualized as changes in cognitive function, mood, and emotional well-being (53). Quality of Life (QoL) was included as an additional category due to its significant role in complementing overall health outcomes in the analyzed population. It encompasses broader dimensions of well-being, including life satisfaction, social participation, and general happiness (54). To avoid confusion, a clear distinction is made between these categories, acknowledging that some outcomes (e.g., life satisfaction) may overlap with both mental health and QoL. This distinction is made to more accurately capture the specific impacts of adaptive cycling interventions on different aspects of health and well-being, allowing for a more comprehensive understanding of their effects.

## 3 Results

Altogether 35 studies were identified, including 34 quantitative and 1 qualitative study, that have investigated the physical and mental health effects of adaptive cycling in individuals with disabilities. The selection process is illustrated in Figure 1 following an adapted form of the PRISMA flow chart (16). All of the studies included in this review concentrate on structured interventions with adaptive cycling, examining the effects of specific cycling interventions on health outcomes in controlled settings.

### 3.1 Overview of the study characteristics

#### 3.1.1 Study characteristics

The following table (Table 4) provides a summary of key variables extracted from the reviewed literature, including sample size, study design, and intervention characteristics. The distribution of studies and their percentages within each variable category is presented. Notably, a wide variance in sample size is evident. The identified literature was categorized into three clusters of study designs: experimental (57.14%), observational (40.00%), and descriptive studies (2.86%). Additionally, the type of intervention was classified to better contextualize the health outcomes of the reviewed studies. This involved categorization into acute interventions (a single session of adaptive cycling), short-term interventions lasting less than 6 months, long-term interventions exceeding 6 months, and investigations examining follow-up effects. The relative share of each intervention type among the 35 articles is as follows: 17.14% for acute interventions, 62.86% for short-term interventions, 17.14% for long-term interventions, and 2.86% for follow-up studies.

**Table 4:** Summary of key variables extracted from the reviewed literature, including sample size, study design, and intervention characteristics.

Variable		
Sample size	Number of subjects	
Minimum	1	
Median	25	
Maximum	162	
	Number of studies	Percentage of studies
Study design		
Experimental studies	20	57.14
Observational studies	14	40.00
Descriptive studies	1	2.86
Intervention		
Acute intervention (single session)	6	17.14
Short-term intervention (<6months)	22	62.86
Long-term intervention (>6months)	6	17.14
Follow-up effect	1	2.86

The table presents the minimum, median, and maximum sample sizes, (number of subjects), as well as the distribution and percentages of studies within the categories of study design and intervention characteristics, based on a total of 35 articles.

Moreover, based on the analysis, the majority of experimental studies focused on short-term interventions (16 studies), followed by acute interventions (2 studies) and long-term interventions (2 studies). Observational studies were predominantly short-term (6 studies), with fewer focusing on acute (4 studies), long-term (3 studies), and follow-up interventions (1 study). Descriptive studies primarily addressed long-term interventions (1 study).

#### 3.1.2 Adaptive cycles

**Figure 2:**
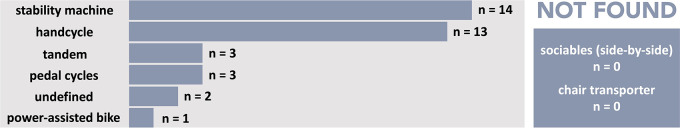
Adaptive cycling modalities identified in the literature search. The figure shows the types of cycling studied for their impact on the physical and mental health of individuals with disabilities, as well as areas lacking research.

The variety of adapted cycles was categorized using the classification suggested by Norcliffe et al. (10). The proposed categories effectively cover the existing types of adaptive cycles and thus allowed for an examination of the available literature. As shown in Figure 2, the highest number of articles were found for stability machines (*n* = 14: 20, 21, 25, 26, 32, 34, 37, 39, 40, 43, 48–51) and handcycles (*n* = 13: 17, 18, 24, 27–29, 33, 35, 36, 44–47). Additionally, three articles were found for pedal (23, 29, 38) and tandem bicycles (30, 31, 41). Two articles could not be assigned to a specific cycle type attributed as undefined (21, 42) and one article was found for power-assisted bikes (19). It is noteworthy that the article of Inckle (29) focused on different types of adaptive cycles, making it assignable to multiple categories. As shown on the right side of Figure 2, no articles were found regarding the mental and physical health impacts of adaptive cycling among individuals with disabilities for the categories sociables (side-by-side), power-assisted bikes, and chair transporters.

For clarity, the identified cycles were categorized as follows: handcycles encompassed all cycles propelled by hand, including arm cycles, indoor handcycles, and traditional handcycles. Stability machines included cycles such as frame runners, stationary bikes, and hip-extensor tricycles. Pedal cycles comprised all adapted cycles with pedals not classified under stability machines, such as recumbent bicycles and tricycles, cycling wheelchairs, trikes, and standard bicycles. Additionally, adaptive cycles were labeled as undefined if the articles did not specify the type of cycle being used.

#### 3.1.3 Population

Within this scoping review, various groups of individuals with disabilities who participated in adaptive cycling interventions were identified. These subject groups encompassed individuals with medical conditions such as spinal cord injuries (17, 18, 33, 35, 36, 45, 47), cerebral palsy (20–22, 26, 28, 34, 38, 39, 42, 51) multiple sclerosis (24), Parkinson's disease (37, 41), and those recovering from acute and chronic strokes (32, 40). Additionally, participants with conditions such as amputations, physical disabilities, impairments, or mobility restrictions, tetraplegia, paraplegia, intellectual and developmental disabilities, multiple trauma histories, and visual impairments (17, 23, 25, 29–31, 35, 46, 49, 50), as reported by the original authors, were also included. Furthermore, other groups of individuals with disabilities were identified that were not clearly categorized by the authors under a specific condition but were described as wheelchair users, frame running athletes, and competitive and recreational handcyclists (27, 43, 44, 48).

### 3.2 Health outcomes of adapted cycling on individuals with disabilities

This section provides an overview of the health effects examined in the selected studies. The outcomes are categorized into physical and mental health effects, and effects on QoL. Within each of the categories, the impact of interventions is discussed based on the duration of the interventions as well as the adaptive cycle used.

#### 3.2.1 Physical health effects

From the examined articles, 27 focused on the physical health effects of adaptive cycling, encompassing various aspects and dimensions of physical performance and health. The acute physical effects of adaptive cycling activities were investigated through handcycling, power-assisted cycling and frame running. Handcycling demonstrated high energy expenditure levels, sufficient to maintain fitness levels and potentially prevent cardiovascular diseases, even when performed at moderate intensity corresponding to 2 mmol/L lactate (17). Similar effects promoting health and fitness adaptations were observed with frame running (39). Moreover, competitive handcyclists exhibited greater flexibility in the thorax (∼5°, *p* < 0.05) and extended their shoulder (∼10°, *p* < 0.01), and posteriorly tilted their scapular (∼15°, *p* < 0.05) more compared to recreational handcyclists suggesting kinematic adaptations attributable to technical training that may optimize muscle recruitment and force generation of the arm (44). Additionally, frame running athletes with neurological motor disorders employed strategies akin to able-bodied runners to absorb impact shock during frame running, positively influencing their movement behavior (43). The use of adapted E-bikes, as shown by Blumenstein et al. (19), can enhance space orientation for individuals with perceptual disorders and allow for the tuning of the electric motor's power to meet individual physical needs, offering tailored support during physical activity.

Regarding the short-term effects of adaptive cycling on physical health, as defined by studies with intervention durations of less than 6 months, a variety of cycles were examined, including handcycles, stability machines, pedal cycles, and tandem cycles. Handcycling interventions, as demonstrated by Bakkum et al. (18) and Gervasoni et al. (24), have shown positive effects on cardiovascular fitness and reductions in fatigue and motor fatigability. Valent et al. (46) and Valent et al. (47) highlighted significant improvements in peak oxygen uptake, mechanical efficiency, and wheelchair capacity associated with handcycle use, including enhancements in arm and shoulder strength and mobility. Similarly, Hoekstra et al. (27) and Kim et al. (33) reported significant improvements in peak oxygen uptake, power output, strength in various muscle groups and body composition parameters following handcycle interventions. These findings collectively underscore the effectiveness of handcycle interventions in promoting both cardiovascular health and muscular strength and suggest their potential in enhancing overall physical well-being.

Using short-term interventions involving stability machines, similar findings were observed. frame running, described by Hjalmarsson et al. (26), contributed to an average increase in cardiorespiratory endurance, accompanied by enhancements in muscle thickness and passive hip flexion, although it was associated with decreased ankle dorsiflexion. As highlighted by Bryant et al. (20), frame running interventions also led to improvements in standing ability and significant improvements of bone quality index scores. Van der Linden et al. (48) highlighted the multiple effects of frame running, including improved muscle stretching, increased self-confidence, and improved functional mobility, although they reported fatigue and muscle soreness. Nevertheless, the latter is a common training adaption of the muscle which improves its strength in the long-term. An intervention with a hip-extensor tricycle by King et al. (34), yielded improvements in gait analysis, although no significant changes were observed in hip extensor strength. Using stationary bicycles, Grecco et al. (25) demonstrated enhancements in overall functional condition, muscle strength, and cardiorespiratory performance. This was further underscored by Fowler et al. (22), who reported substantial improvements in locomotor endurance, gross motor function, and strength. Additionally, individuals with disabilities using stationary bicycles were observed to experience additional enhancements in balance, mobility, and gait abilities, along with improvements in activities of daily living (32, 37). Overall, these findings emphasize the positive impact of short-term interventions involving stability machines in enhancing cardiorespiratory endurance, strength and functional mobility, thereby supporting improvements in overall physical health.

Pedal cycles, including cycling wheelchairs, bicycles, and tricycles, have also demonstrated substantial benefits in various physical health aspects, particularly through short-term interventions. Interventions with cycling wheelchairs resulted in improvements in aerobic capacity (23), while interventions involving bicycles and tricycles significantly enhanced locomotor performance, lying, and reversal motor capacities (38). Following a tandem cycling intervention, McGough et al. (41) observed statistically significant improvements in physical performance across gait, balance, and mobility domains.

In addition, two short-term interventions with undefined adaptive cycles found that significant improvements in the energy expenditure index as well as gross motor function can be achieved (21) and improvements in functional ability were associated with adapted cycles (51).

While the findings of short-term interventions collectively underscore the diverse and significant positive impacts of adaptive cycling on physical health and function, it is equally important to consider the long-term effects of interventions lasting more than 6 months to fully understand the potential of adaptive cycling on physical health for individuals with disabilities. Altogether five studies were found that investigated the long-term effects including handcycles, tandem cycles and stability machines. Handcycling led to significant improvements in arm swing and flexion angles during the gait cycle (28), with a notable increase in physiological parameters among paraplegic individuals, while its influence post-rehabilitation was inconclusive (45). Tandem cycling resulted in statistically significant increases in VO_2_max and Pmax, with elite cyclists exhibiting significantly higher VO_2_max compared to sub-elite cyclists (30); additionally, it demonstrated similar improvements in hemodynamic variables (e.g., heart rate, stroke volume, cardiac output, ejection fraction, systemic vascular resistance), suggesting that severe visual impairment does not impede the health benefits of regular physical activity (31). Following a long-term intervention with stationary bicycles, Mayo et al. (40), found that stationary cycling was equally effective in maintaining walking capacity after discharge from stroke rehabilitation as were mobility exercises and brisk walking.

#### 3.2.2 Mental health effects

Seven articles addressed the mental health effects of adaptive cycling among individuals with disabilities. Vogt et al. (50) found that acute stationary bicycle use temporarily enhances neuronal activity, particularly in relation to cognitive performance in individuals with intellectual and developmental disabilities. Short-term effects were observed across a restricted range of cycling modalities. Frame runner and hip-extensor tricycle interventions were associated with increased enjoyment (20, 34) and increased self-confidence (48), while adaptive bicycle use was associated with an improved sense of well-being (42). Handcycling was linked to increased life satisfaction during the training period, although no significant changes in mental health were noted over time (35). When examining the long-term effects studied by Inckle (29), a perceived enhancement in the experience of mobility, independence, and freedom, along with significant benefits for mental and physical health and wellbeing of the individuals involved, can be observed.

#### 3.2.3 Effects on quality of life

In addition to the physical and mental health benefits, five studies also examined changes in QoL among individuals with disabilities throughout adaptive cycling interventions. Fu et al. (23) reported enhancements in QoL using cycling wheelchairs among elderly with physical disability. Similarly, frame running interventions have been associated with positive changes in various aspects of QoL, as reported by parents of participating children. These changes were particularly evident in performance, participation ability, happiness, and self-confidence, suggesting an overall increase in QoL (49). In contrast, Bryant et al. (20) found an increase in enjoyment using frame runner but no changes in QoL. Lauhoff et al. (37) supported those findings, observing no significant impact on QoL following a stationary bicycle use.

Follow-up examinations of handcycle interventions conversely revealed significant changes in body satisfaction. Body satisfaction significantly increased during the training period but decreased back to pre-training levels at follow-up, whereby individuals with more severe impairments exhibited a larger decrease in body satisfaction. Additionally, improvements in physical capacity and waist circumference were significantly associated with improvements in body satisfaction (36).

## 4 Discussion

The present scoping review aimed to comprehensively examine the existing literature on the physical and mental health effects of adaptive cycling in individuals with disabilities. While the review identified a substantial body of research investigating the effects of various adaptive cycling interventions, several noteworthy gaps and future research directions emerged from the analysis.

Adaptive cycling interventions demonstrated significant potential for enhancing physical health outcomes, including improvements in cardiovascular fitness, muscle strength, mobility, and functional capacity. Specifically, interventions involving handcycles, stability machines, pedal cycles, and tandem cycles were associated with positive changes in physical fitness parameters such as peak oxygen uptake, power output, and muscle strength. Moreover, adaptive cycling activities promoted cardiovascular health, with studies indicating improvements in cardiorespiratory endurance and energy expenditure levels. In terms of mental health, a limited number of studies addressed this aspect, but those available found positive effects. Short-term effects on mental well-being were observed across various cycling modalities, including increased enjoyment and a sense of well-being. However, the evidence base for mental health outcomes was less extensive compared to physical health outcomes, indicating a need for further research in this area.

Controversies and inconsistencies were also observed, particularly regarding QoL outcomes. While some studies reported significant enhancements in QoL domains, such as performance, participation ability, happiness, and self-confidence, others found no effects of adaptive cycling on QoL. These discrepancies underscore the need for further research to elucidate the true impact of adaptive cycling on QoL outcomes and to address potential confounding factors that may influence individual perceptions.

Furthermore, there was variability in the quantity and quality of evidence across different categories of adaptive cycling modalities. While certain modalities, such as handcycles and stability machines, were supported by a relatively robust body of evidence, others, such as sociable cycles, chair transporters, and power-assisted bikes, were underrepresented or lacked sufficient empirical support. This underscores the necessity for further research to investigate the diverse array of adaptive cycling options and their effects on health outcomes among individuals with disabilities, particularly as those alternative cycling modalities show potential but have not been adequately studied. Future research should explore their potential benefits and feasibility to provide a more comprehensive understanding of adaptive cycling options.

Moreover, it is essential to consider the broader context of adaptive physical activity. Research on other forms of physical activity, such as adapted sports and exercise interventions for individuals with disabilities, has shown similar benefits in terms of physical and mental health, as well as QoL (55–57). This review's findings align with the broader literature on adaptive physical activity, underscoring the importance of structured and inclusive physical exercise for individuals with disabilities. Future studies should explore how adaptive cycling may complement or differ from other adaptive physical activities, contributing to a more comprehensive understanding of the role of physical activity in improving overall health outcomes.

Another significant issue in the existing literature is the inconsistency in how populations are defined and categorized. While various groups of individuals with disabilities have participated in adaptive cycling interventions, the studies often lack clear and standardized definitions of these populations. For instance, some studies include individuals with spinal cord injuries, cerebral palsy, multiple sclerosis, and other specific conditions, while others broadly categorize participants as wheelchair users or individuals with physical disabilities. This lack of uniformity in population categorization limits the ability to compare and generalize findings across studies. Moreover, some disability groups, such as those with intellectual and developmental disabilities or sensory impairments, remain underrepresented. Additionally, gaps remain in assessing health outcomes related to the community usage of adaptive cycling, which could provide valuable insights into its real-world impact and broader applicability. Future research should aim to standardize the categorization of disability populations and ensure inclusivity, also in relation to community settings, to better capture the diverse needs and experiences of individuals with disabilities.

The studies reviewed in this scoping review varied considerably in terms of sample size, study design, and intervention duration, highlighting challenges in interpreting the findings. The wide variance in sample sizes, with some studies involving very small participant groups, may impact the reliability and generalizability of the results. Additionally, while adaptive cycling shows potential for improving physical and mental health, the predominance of short-term interventions limits our understanding of its long-term effects, underscoring the need for further research with larger sample sizes and extended intervention and follow-up periods.

### 4.1 Limitations

While every effort was made to conduct a comprehensive search of the literature, it is always possible that some studies were not captured by the search terms. Despite trying alternative search criteria, it is always possible that mis-specified keywords and inaccurate abstracts led to the exclusion of certain studies that could have provided further insights into the topic. This review included studies published in English and German to reflect the authors linguistic abilities; studies in other languages were therefore not included, which may have limited representation from regions where research is predominantly published in other languages. Furthermore, the quality and heterogeneity of the included studies may have influenced the synthesis and interpretation of the findings. Although we did not develop a formal protocol or conduct a critical appraisal—given that our aim was to map the breadth of the existing literature rather than evaluate study quality—we adhered to the remaining PRISMA-ScR guidelines to ensure a comprehensive and transparent report of our methods and findings.

## 5 Conclusion

In summary, this scoping review consolidates the current evidence on the physical and mental health effects of adaptive cycling for individuals with disabilities, and emphasises significant gaps in the literature. The findings demonstrate the potential of adaptive cycling to enhance physical health, mental health, and quality of life. However, inconsistencies in the evidence, especially concerning quality-of-life outcomes, highlight the need for further investigation.

Beyond summarizing existing evidence, this review underscores the importance of expanding research to include underrepresented populations and modalities, such as sociable cycles and chair transporters. Future research should also explore the long-term effects of adaptive cycling interventions and consider the broader spectrum of health outcomes associated with various cycling modalities over different time periods. Such efforts could provide a more comprehensive understanding of adaptive cycling's benefits and ensure its accessibility for diverse disability groups.

Ultimately, this review serves as a foundation for future research and practice in the field of adaptive cycling for individuals with disabilities. Its findings have the potential to inform clinical decision-making, guide future research endeavors, and thereby foster improved health and well-being of individuals with disabilities.

## Data Availability

The original contributions presented in the study are included in the article/Supplementary Material, further inquiries can be directed to the corresponding author.
